# Impact of Intrapericardial Fluid on Lesion Size During Epicardial Radiofrequency Ablation: A Computational Study

**DOI:** 10.3390/jcdd12080283

**Published:** 2025-07-24

**Authors:** Luis Cuenca-Dacal, Marcela Mercado-Montoya, Tatiana Gómez-Bustamante, Enrique Berjano, Maite Izquierdo, José M. Lozano, Juan J. Pérez, Ana González-Suárez

**Affiliations:** 1 BioMIT, Department of Electronic Engineering, Universitat Politècnica de València, Building 7F, Camino de Vera, 46022 Valencia, Spain; luis.cuencadacal@gmail.com (L.C.-D.); eberjano@upv.es (E.B.); jjperez@eln.upv.es (J.J.P.); 2 Silico STEM S.A.S., Medellín 050035, Colombia; marcela.mercadom@insilicostem.com (M.M.-M.); tatiana.gomez@insilicostem.com (T.G.-B.); 3 Electrophysiology Section, Cardiology Department, Hospital Universitario y Politécnico La Fe, 46026 Valencia, Spain; maiteizqui@hotmail.com; 4 Arrhythmias Unit, Cardiology Department, Hospital Universitario Clínico San Cecilio, 18016 Granada, Spain; jlozanoherrera62@gmail.com

**Keywords:** computer modeling, epicardial ablation, in silico model, lung, radiofrequency ablation, ventricular tachyarrhythmia

## Abstract

Background and aims: Epicardial RFA is often required when ventricular tachyarrhythmias originate from epicardial or subepicardial substrates that cannot be effectively ablated endocardially. Our objective was to evaluate the impact of intrapericardial fluid accumulation on the lesion size in the myocardium and the extent of thermal damage to adjacent structures, particularly the lung. Methods: An in silico model of epicardial RFA was developed, featuring an irrigated-tip catheter placed horizontally on the epicardium. A 50 W–30 s RF pulse was simulated. Temperature distributions and resultant thermal lesions in both the myocardium and lung were computed. Results: An increase in pericardial space from 2.5 mm to 4.5 mm resulted in a reduction of myocardial lesion depth by up to 1 mm, while the volume of lung damage decreased from 200 to 300 mm^3^ to nearly zero, irrespective of myocardial or epicardial fat thickness. Myocardial lesion size was markedly influenced by the thickness of the epicardial fat layer. In the absence of fat and with a narrow pericardial space, lesions reached up to 262 mm^3^ in volume and 6.1 mm in depth. With 1 mm of fat, lesion volume decreased to below 100 mm^3^ and depth to 3 mm; with 2 mm, to under 40 mm^3^ and 2 mm; and with 3 mm, to less than 16 mm^3^ and 1.2 mm. Lung damage increased moderately with greater fat thickness. Cooling the irrigation fluid from 37 °C to 5 °C reduced lung damage by up to 51%, while myocardial lesion size decreased by only 15%. Conclusions: Intrapericardial fluid accumulation can limit myocardial lesion formation while protecting adjacent structures. Cooling the irrigation fluid may reduce collateral damage without compromising myocardial lesion depth.

## 1. Introduction

**Figure 1:**
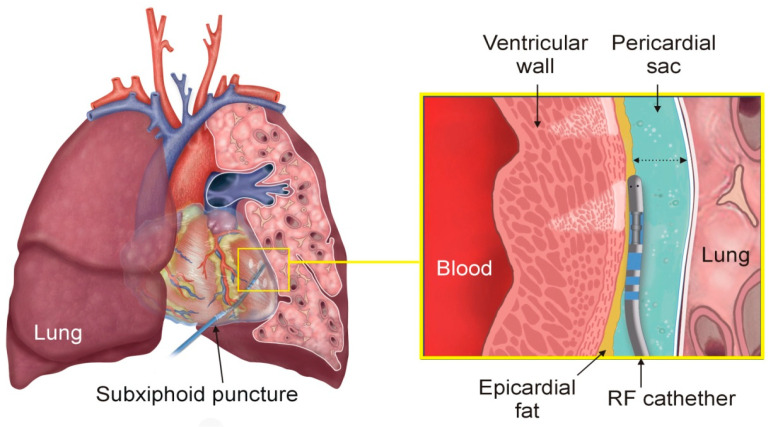
Physical situation during epicardial RF ablation of ventricular tachyarrhythmia: The illustration highlights the positioning of the heart between the lungs, with a portion of the left lung removed to allow visualization of the RF catheter in the epicardial region (**Left**). A detailed view of the RF catheter within the pericardial sac is shown, highlighting the surrounding tissues: epicardial fat, myocardium, intracavitary blood, and lung (**Right**). The dotted arrow represents the pericardial space.

Epicardial radiofrequency (RF) ablation (RFA) has proven to be an effective treatment for ventricular tachyarrhythmia (VT) in an unsuccessful endocardial RFA due to the presence of epicardial and subepicardial arrhythmogenic substrates. The subxiphoid puncture approach is currently regarded as the gold standard procedure for performing the epicardial RFA [1,2,3,4]. This technique involves positioning the RF catheter within the pericardial sac over the ventricles in close proximity to other structures such as the left lung, as illustrated in Figure 1. Irrigated-tip catheters are commonly used for these procedures. Unlike endocardial ablation, where the irrigation fluid is mixed with and drained by the bloodstream, the irrigation fluid used in epicardial procedures accumulates within the pericardial space, which causes the sac to expand, requiring manual drainage multiple times throughout the procedure [5].

Preclinical studies have studied the performance of RF catheters for epicardial RFA by examining lesion characteristics and the influence of different variables, including the presence of visceral fat [6,7], the use of normal saline (NS) vs. half-normal saline (HNS) as irrigation fluid [7], contact force [7,8,9,10], power settings [10,11], irrigation flow rate and intrapericardial fluid accumulation [5], proximity to the coronary arteries [12], unipolar vs. bipolar ablation [13], closed-loop vs. open irrigation cooling [14] and damage to adjacent structures [15].

Although in silico studies have also examined RF lesion size during epicardial RFA [16,17,18], to the best of our knowledge, no previous in silico studies have evaluated how the volume of intrapericardial fluid accumulated into the pericardial sac affects lesion formation in both the myocardium and adjacent structures, such as the lung. Moreover, the potential impact of the irrigated fluid temperature on lesion characteristics has still not been explored. In this context, computational modeling provides significant advantages for studying thermal distribution in tissues under controlled conditions, enabling precise and simultaneous control over variables that are difficult to isolate in preclinical experimental models, such as ventricular wall thickness, the presence of epicardial visceral fat, and the pericardial space width (resulting from intrapericardial fluid accumulation). Our objective was to study the impact of irrigation fluid accumulation on both myocardial lesion formation and thermal damage to adjacent structures (lung) during RF pulse delivery to the epicardium. We used computer simulation and considered various anatomical and procedural factors, such as the presence of epicardial fat, myocardial wall thickness, and the temperature of the irrigated fluid.

## 2. Methods

### 2.1. Model Geometry

**Figure 2:**
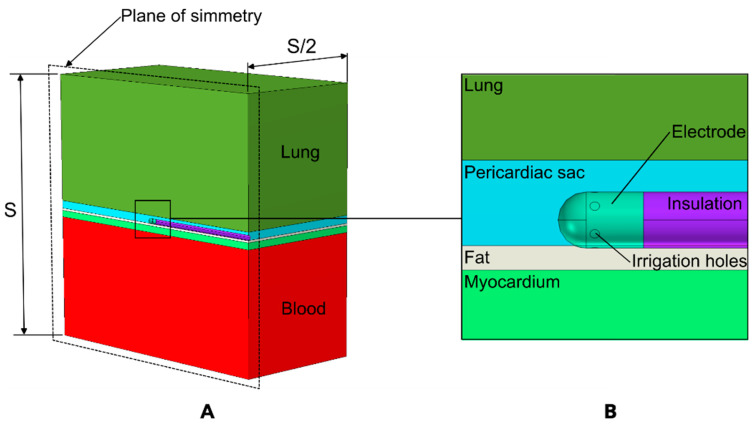
(**A**) Overview of the geometry of the computational model, displaying only half of the model due to the symmetry plane (S = 120 mm). (**B**) Detailed view around the ablation electrode.

A three-dimensional model was developed to simulate the physical scenario depicted in Figure 1. Figure 2 shows the model geometry, which includes a fragment of ventricular wall, the RF catheter in contact with the epicardium, intracavitary blood within the ventricle, a portion of pericardial sac containing the irrigation fluid and the RF catheter, and a fragment of lung. The RF catheter was assumed to be in horizontal contact with the surface of the epicardium, as this is the most commonly used position in clinical practice [3,4] and to slightly deform the cardiac tissue to a depth of 0.2 mm to ensure continuous contact during RF energy delivery. The lung lies beyond the pericardial sac. The model included a symmetry plane, which allowed us to consider only half of the model, thereby reducing the computational cost.

The myocardial wall thickness was assumed to range from 3 to 15 mm [19,20], the visceral fat varied from 0 (no fat) to 3 mm, and the pericardial space width (PSW) ranged from 2.5 to 4.5 mm. These ranges allow for a sufficiently large set of simulations (a total of 100) to capture the variability found in clinical practice. The modeled irrigated tip was of an RF catheter, TactiCath™ (Abbott Laboratories, Abbott Park, IL, USA). The electrode was modeled as a sheet metal, considered hollow with a wall thickness of 0.2 mm and a rounded tip of 3.5 mm in length and 2.3 mm (7 Fr) in diameter. It featured six evenly spaced 0.4 mm diameter irrigation holes. The model also included the insulated portion of the catheter, which was tube-shaped with an annular cross-section and a thickness of 0.65 mm.

### 2.2. Underlying Biophysics

The Supplementary Material describes the governing equations and the boundary conditions associated with the biophysics problems solved, which include electromagnetic heating, heat transfer and fluid dynamics. These three problems were coupled and solved using the Finite Element Method in COMSOL Multiphysics^®^ software 6.2 (COMSOL, Burlington, MA, USA). Electromagnetic heating was modeled by Laplace’s Equation. An RF pulse of 50 W for 30 s with a flow rate of 34 mL/min was considered, which is a common setting in these procedures [21,22,23]. Fluid mechanics was modeled by the Navier–Stokes equations, assuming constant viscosity, incompressible flow and negligible effects of gravity. The temperature distribution was computed using the Bioheat Equation [24], which incorporated the thermal sink effect due to capillary blood perfusion. The transient simulations also included a 90 s period following the RF pulse to account for the effects of thermal latency and their impact on additional lesion growth [25]. Thermal damage to the myocardium and lung tissues was evaluated using the Arrhenius model, which offers a quantitative description of tissue damage based on the cumulative effect of time and temperature [24] (for further details, see Supplementary Materials).

Complementary simulations were conducted by varying the temperature of the irrigated saline over a wide range, from 5 to 37 °C, to assess its potential effect on the myocardium and lung lesions. For the rest of the simulations we considered a temperature of 21 °C as it exited the electrode, assuming that the saline does not experience any heating during its path from the bag (at room temperature) to the catheter tip.

### 2.3. Properties of Materials and Tissues

Table 1 shows the physical properties of the model elements. The thermal conductivity, density, specific heat and electrical conductivity (at 500 kHz and 37 °C) of biological tissues (myocardium, blood, visceral fat, and lung) were obtained from the IT’IS Foundation database [26]. The properties of the irrigation fluid were obtained from [27]. The electrical conductivity of tissue and fluid increased by 1.5%/°C in myocardium, fat, and lung [28], and by 2%/°C in blood and the irrigation fluid [28,29]. When the temperature reached 100 °C, the electrical conductivity dropped by two orders of magnitude to model the water loss associated with vaporization. The extra energy required for the phase change (latent heat) was modeled by increasing the specific heat by a factor of 400 within the 100–101 °C range [28]. The dynamic viscosity of blood was obtained from [30], and the viscosity of the irrigation fluid was assumed to be the same as that of water, as reported in [27].

## 3. Results

### 3.1. Overview of the Thermal and Electrical Performance

**Figure 3:**
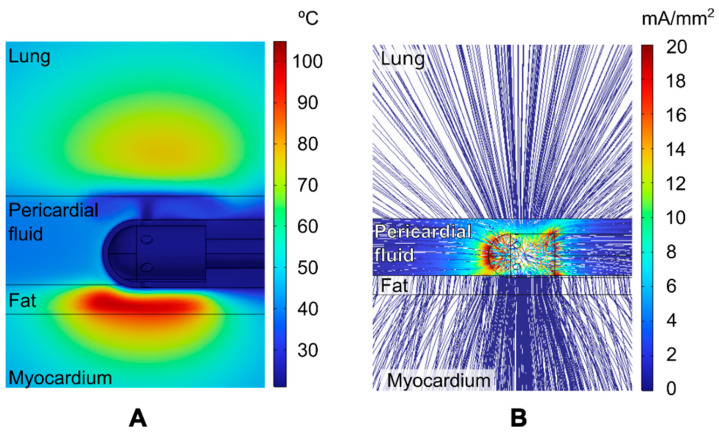
Temperature distribution (**A**) and streamline diagram of the current density (**B**) at the end of a 30 s 50 W RF pulse (case of 1 mm fat, 8 mm cardiac wall and 3 mm pericardial space width). Color scale in °C in plot (**A**) and mA/mm^2^ in plot (**B**) (with the range clipped between 0 and 20 mA/mm^2^ for clarity, reaching a maximum value of 188 mA/mm^2^ at the tip of the electrode and at the junction between electrode and plastic tube).

Figure 3 shows the distribution of temperature and current density around the RF electrode for the particular case of 1 mm of epicardial fat, an 8 mm myocardial wall, and a 3 mm PSW. This case is representative as it is based on parameter values that are approximately at the midpoint of the ranges considered in this study. The highest temperature was reached in the epicardial fat (~100 °C), while the maximum temperature in the lung remained below 78 °C (see Figure 3A). The thermal effect of the irrigation jet in the outlet zones is highly noticeable, contributing to the maintenance of a relatively consistent cool temperature within the pericardial sac. The cooling effect prevents the fat surface from reaching high temperatures, despite the high temperature just 0.5 mm below.

**Figure 4:**
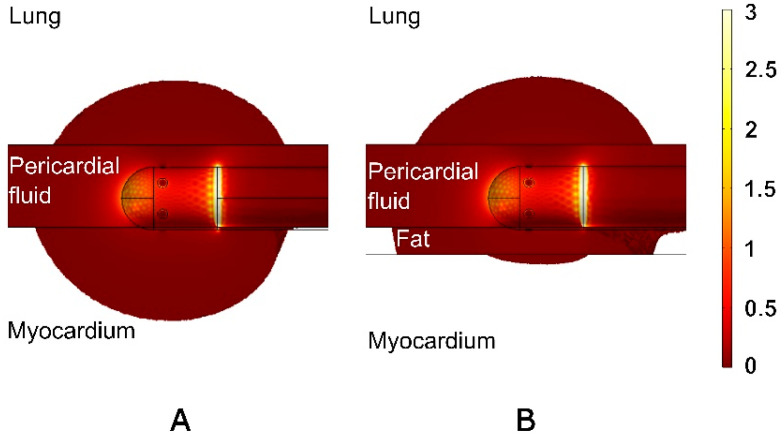
RF power density (in W/mm^3^) around the ablation electrode without (**A**) and with 1 mm of fat (**B**) in the case of 8 mm cardiac wall and 3 mm pericardial space width. The range was clipped for clarity between 0.005 and 3 W/mm^3^, with a maximum value of 19.7 W/mm^3^, specifically at the junction between the electrode and the plastic tube.

Figure 3B clearly shows that RF current preferentially flows through the intrapericardial fluid. It is also evident that the current density in the lung near the RF electrode is comparable to that in the myocardium directly beneath the electrode. Therefore, it is reasonable to assume that if the RF current induces a lesion in the myocardium, it could theoretically also induce a lesion in the lung. Figure 4 shows the RF power density (in W/mm^3^) (also known as resistive heating) around the ablation electrode for the case of an 8 mm cardiac wall and a 3 mm PSW, both with and without fat and with a 1 mm fat layer. Most of the RF power is deposited in the intrapericardial fluid as it practically surrounds the entire electrode. It should be noted that the RF power deposited in the lung, despite being at a certain distance from the electrode, is not negligible for both cases with and without fat. It is also noteworthy that the resistive heating in the myocardium is significant in the absence of fat (see Figure 4A) but considerably reduced in the case of 1 mm fat, as the RF power is mainly deposited in the fat itself.

### 3.2. Evolution of Lesion Volume in Myocardium and Lung

**Figure 5:**
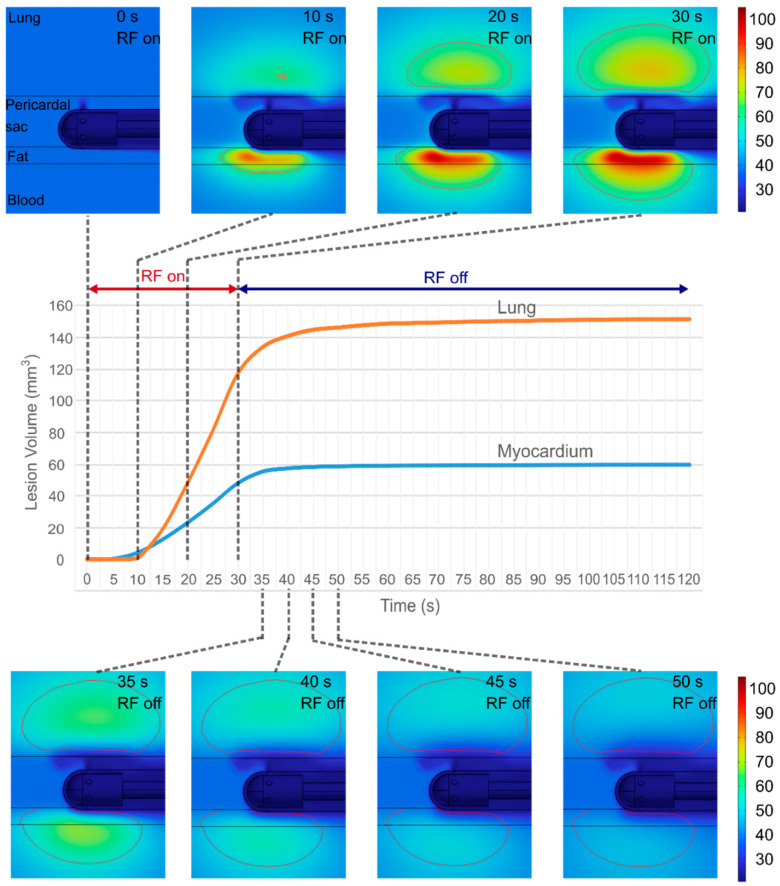
Temperature distributions during the RF pulse (30 s) and the subsequent period (scale in °C), and evolution of the total damaged tissue (in lung and myocardium, excluding epicardial fat). The red solid line represents the thermal lesion boundary computed using the Arrhenius model to estimate accumulated damage (case of 1 mm fat, 8 mm cardiac wall and 3 mm pericardial space width of 3 mm).

Figure 5 shows the evolution of the temperature distributions around the electrode and the lesion geometry during the 30 s RF pulse and the post-RF period (90 s) for the case of 1 mm fat, 8 mm cardiac wall and 3 mm PSW. Heating is primarily concentrated in the myocardium and fat. No thermal damage is seen in either the myocardium or the lung in the first 5 s. Although myocardial damage is initially more pronounced (during the first 10 s), lung damage progresses at a faster rate, despite experiencing lower temperatures than the myocardium and fat, so that the total lesion volume in the lung is 154% larger (151 mm^3^ vs. 59 mm^3^). The lesion extends to a depth of 5 mm in the lung, compared to 2.6 mm in the myocardium. Interestingly, the lung lesion is slightly displaced inward, indicating that it does not make direct contact with the surface of the pericardial sac.

### 3.3. Effect of the Anatomical Parameters on Myocardium and Lung Lesion

**Figure 6:**
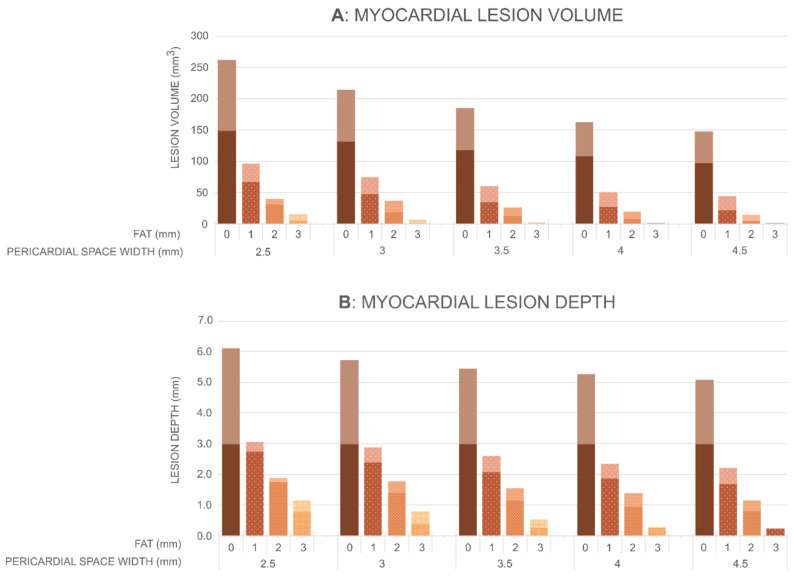
Myocardial lesion volume and depth for different thicknesses of epicardial fat (0, 1, 2, and 3 mm) and widths of pericardial sac (2.5, 3, 3.5, 4, and 4.5 mm). On each bar, the lower segment indicates the minimum value, and the upper segment indicates the maximum value across the different myocardial thicknesses (3, 5, 8, 11, and 15 mm).

**Figure 7:**
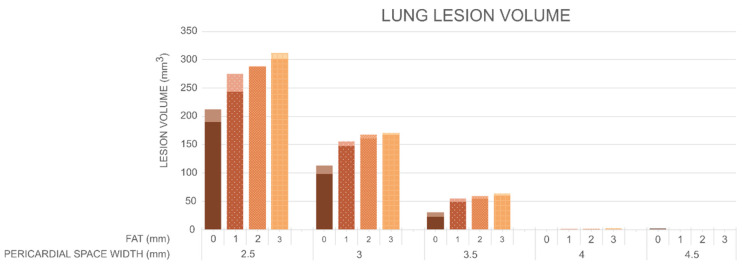
Lung lesion volume (in mm^3^) for different thicknesses of epicardial fat (0, 1, 2, and 3 mm) and pericardial sac (2.5, 3, 3.5, 4, and 4.5 mm). On each bar, the lower segment indicates the minimum value, and the upper segment indicates the maximum value across the different myocardial thicknesses (3, 5, 8, 11, and 15 mm).

Figure 6 shows the myocardial lesion volumes and depths, while Figure 7 shows the lung lesion volumes calculated by the Arrhenius model for different values of PSW (2.5, 3, 3.5, 4 and 4.5 mm), epicardial fat thicknesses (0, 1, 2 and 3 mm), and cardiac wall thicknesses (3, 5, 8, 11 and 15 mm). The first key finding is that the pericardial space has a clear impact on lesion formation in the myocardium and thermal damage in the lung, although the magnitude of this effect differs between tissues. In the myocardium, an increase in the pericardial space leads to a reduction in both lesion volume and depth (as long as the lesion is not transmural). For instance, in the absence of epicardial fat and with a myocardium thickness of 8 mm, the lesion volume decreases from 262 mm^3^ at 2.5 mm of pericardial space to 130 mm^3^ at 4.5 mm (a 50% reduction), and the lesion depth decreases from 6.1 mm to 5.1 mm. Similar trends are observed for thicker myocardium. For example, for a myocardium thickness of 15 mm, the lesion depth decreases from 5.5 mm to 4.5 mm as pericardial sac width increases from 2.5 mm to 4.5 mm. The effect is more pronounced when epicardial fat is present. With 2 mm of epicardial fat and a myocardium thickness of 8 mm, the lesion volume decreases from 40 mm^3^ to 8 mm^3^ (an 80% reduction), and the depth drops from 1.9 mm to 0.9 mm over the same range of PSW. The impact of pericardial space is even more pronounced in the lung. Across all tested configurations, including those with epicardial fat, both lesion volume and depth in the lung decrease sharply as the pericardial space increases. For instance, in the absence of epicardial fat and with a myocardium thickness of 5 mm, the lesion volume decreases from 193 mm^3^ at 2.5 mm of pericardial space to 0 mm^3^ at widths over 4 mm, while the lesion depth drops from 8.2 mm to zero. A similar trend is observed when 1 mm of epicardial fat is present: the lesion volume decreases from 275 mm^3^ with 2.5 mm of pericardial space, to 0 mm^3^ with pericardial space over 4 mm, and the lesion depth is reduced from 6.2 mm to zero. These findings highlight the strong protective effect of intrapericardial fluid in “shielding” adjacent tissues such as the lung from thermal damage, with even relatively thin layers (≥4 mm) being sufficient to completely prevent lesion formation in this region.

The second finding is that epicardial fat thickness has a strong influence on the lesion size created in the myocardium, with a more limited effect on the lung damage volume. As fat thickness increases, the myocardium lesion size decreases for all the considered values of myocardium thickness and pericardial space. In particular, for a pericardial space of 2.5 mm, and depending on myocardial thickness, the lesion volume in the myocardium ranges from 149 to 262 mm^3^ in the absence of epicardial fat and decreases progressively with increasing fat thickness: falling below 100 mm^3^ with 1 mm of fat, to approximately 40 mm^3^ with 2 mm of fat, and to 16 mm^3^ with 3 mm of fat. A similar trend is observed for lesion depth, which ranges from 5.1 to 6.1 mm with no fat, decreases to 2.2–3.1 mm with 1 mm of fat, 1.2–1.9 mm with 2 mm, and 0.3–1.2 mm with 3 mm of fat. The trend is opposite in terms of thermal damage to the lung, with a smaller impact: as the thickness of epicardial fat increases, lesion volume increases from approximately 200 mm^3^ for the case without fat to more than 300 mm^3^ in the case of 3 mm fat (values in the case of a PSW of 2.5 mm).

Finally, myocardial thickness was found to have a limited influence on both lesion size and the extent of thermal damage to the lung. As myocardial thickness increased, myocardial lesion size tended to decrease, whereas thermal damage to the lung exhibited a slight increase. These trends were observed after excluding simulations in which transmural lesions were achieved. Although consistent, the observed differences were modest. In the myocardium, lesion depth varied by approximately 0.5 mm, while changes in lung damage were even smaller.

### 3.4. Effect of Irrigation Fluid Temperature

**Figure 8:**
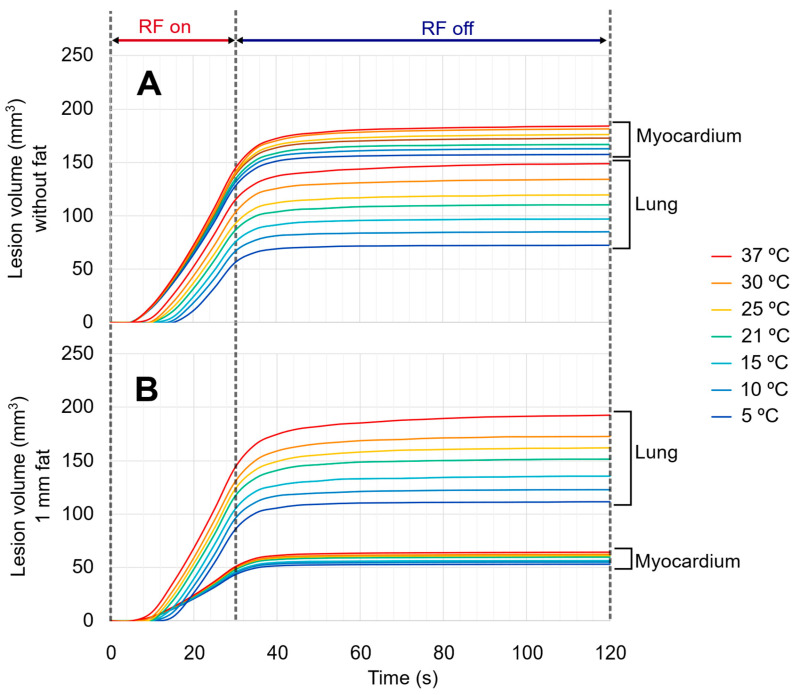
Evolution of the damaged tissue (in lung and myocardium, excluding epicardial fat) for different values of the irrigation saline temperature, from 5 to 37 °C, without fat (**A**) and in the case of 1 mm fat (**B**) (representative case of 8 mm cardiac wall and 3 mm pericardial space width).

Figure 8 shows the evolution of the total damaged tissue in the myocardium and lung (excluding epicardial fat) for different irrigation fluid temperatures, comparing two scenarios: pulses over the myocardium without epicardial fat and with 1 mm of epicardial fat. In both cases, the cardiac wall thickness was 8 mm and the pericardial space was 3 mm. While the irrigation fluid temperature had a limited effect on myocardial damage, especially in the presence of epicardial fat, its impact on lung damage was considerably more pronounced. In the absence of epicardial fat, myocardial damage decreased moderately when the temperature of the irrigated saline was reduced, specifically from 184 mm^3^ with 37 °C to 157 mm^3^ with 5 °C. This represents a reduction of approximately 15% in myocardial damage when using cold irrigation compared to body temperature irrigation. Notably, lesion depth in the myocardium remained constant at 4.6 mm across all irrigation temperatures. By contrast, the impact on the lung was substantially greater, with damage decreasing from 149 mm^3^ to 72 mm^3^ over the above-mentioned temperature range, which corresponds to a 51% reduction. When 1 mm of epicardial fat is present, the influence of irrigation fluid temperature on myocardial lesion is also limited (from 64 mm^3^ to 52 mm^3^, representing a reduction of approximately 17%), and lesion depth remains stable at 2.6 mm. In contrast, lung damage still shows a marked drop, from 193 mm^3^ with 37 °C to 111 mm^3^ with 5 °C, corresponding to a 42% reduction.

## 4. Discussion

This study presents a computational model developed to analyze lesion size during epicardial RFA in the ventricle. The model includes the main tissues and surrounding media involved in epicardial RFA (myocardium, epicardial fat, pericardial fluid, blood, and lung) as well as key physical features of the irrigated-tip RF catheter. This study focuses on understanding how intrapericardial fluid accumulation influences lesion formation during epicardial RFA on the myocardium and additional consideration of effects on nearby tissues such as the lung. To better understand this relationship and account for the anatomical variability encountered in clinical practice, we analyzed a wide range of simulated scenarios. Specifically, we varied the pericardial space, which represents different degrees of pericardial “distension” caused by fluid accumulation, and evaluated its impact on lesion size across combinations of myocardial thickness and epicardial fat. This approach allows us to isolate the role of pericardial fluid while considering relevant anatomical factors that may modulate its effect.

### 4.1. Impact of Pericardial Space on the Myocardial Lesion Size

Myocardial lesion volume and depth tend to decrease with increasing intrapericardial fluid volume. For instance, a difference of approximately 1 mm in lesion depth was observed between simulations with pericardial spaces of 2.5 mm and 4.5 mm. This effect is likely explained by the conductive nature of the irrigated saline, which carries a substantial portion of the RF current and thus becomes the main site of energy deposition during the pulse, reducing the amount of energy that reaches the myocardium (Figure 3B). These findings align with those of Aryana et al. [5], who reported smaller lesions in the myocardium in the presence of a significant amount of intrapericardial fluid during epicardial RFA in an experimental animal model. While their study evaluated only two levels of fluid accumulation, our computational model explores a wider and more detailed range of intrapericardial fluid volumes, enabling a more comprehensive assessment of its impact on lesion formation. Moreover, our simulations allow quantification of the energy deposited in each tissue and in the conductive media traversed by the RF current, offering further insight into the mechanisms underlying lesion formation and collateral damage.

The influence of fluid conductivity might also help explain why using irrigation fluids with lower conductivity, such as Half Normal Saline (HNS), has been reported to increase lesion size [7], and it also raises the possibility that HNS might impact thermal damage to surrounding tissues, although this effect has yet to be thoroughly examined.

### 4.2. Impact of Pericardial Space on the Heating in the Lung

Concerning the impact on surrounding tissues, our results indicate that under certain conditions, a small volume of the lung adjacent to the ablation site may also be thermally damaged. Specifically, for the simulated energy setting (50 W for 30 s), pericardial spaces smaller than 4 mm in width increased the likelihood of damage in the lung, regardless of myocardial wall thickness (3–15 mm) or fat thickness (0–3 mm), as shown in Figure 7. In addition, the progress of the thermal lesion in the myocardium and lung seems to follow very different dynamics. While lesion formation in the myocardium initiates almost immediately at the onset of the RF pulse and attains very high temperatures (approximately 100 °C) by the end of the pulse, thermal lesions in the lung manifest with a delay of approximately 10 s and are associated with lower temperature elevations (see Figure 5). This different behavior is likely due to the relative positioning of the ablation electrode in relation to the myocardium and lung. The ablation electrode is in firm contact with the cardiac wall (with or without the presence of fat), allowing for direct RF energy transfer, while the irrigation holes are partially sealed by the tissue itself, promoting rapid resistive heating in that place. On the other hand, the ablation electrode is slightly separated from the lung by the fluid occupying the pericardial sac, which consists primarily of irrigated saline during ablation. This separation prevents the direct deposition of RF power into the lung, minimizing resistive heating during the first few seconds of ablation. Additionally, the specific positioning of the irrigation holes, as shown in Figure 3A, could facilitate active cooling of the lung area through the irrigated saline jet. Note that this potentially advantageous positioning of the irrigation holes relative to the tissue surface, in terms of minimizing thermal damage to the lung, is inherent in the horizontal position of the RF catheter, required by the anatomy and access to the pericardial sac.

However, thermal lesion in the lung develops gradually throughout the RF pulse and continues to form even after the pulse has ended. This occurs despite the fluid inside the pericardial sac remaining at a relatively steady cold temperature during ablation (see Figure 5), which acts as a heat sink. Our physical explanation for this is that although there is no direct contact between the RF electrode and the lung, a certain amount of RF energy is actually deposited in the lung, as suggested by the power density in the lung region near the RF electrode (see Figure 4).

At the end of the RF pulse, the temperature in the myocardium drops rapidly due to the cooling effect of the irrigating saline and in part due to the cooling provided by circulating blood in the cardiac chamber. In contrast, these cooling mechanisms seem to have less impact in the lung. The greater thermal conductivity of the myocardium than that of the lung (0.56 vs. 0.39 W/K·m) probably helps to reduce the temperature in the myocardium, which seems to be a counterintuitive result: the total volume of damaged tissue is greater in the lung despite the temperatures in the lung being lower than those in the myocardium.

It could be argued that the heating in the lung develops more slowly than in the myocardium. Also, the fact that the lung does not reach 100 °C may also contribute to the continued flow of RF current through it, unlike in the myocardium, where temperatures near 100 °C cause significant tissue desiccation and a sharp reduction in electrical conductivity.

### 4.3. Effect of Irrigation Fluid Temperature

Finally, we found that the temperature of the irrigation fluid may influence thermal damage to structures adjacent to the myocardium without compromising lesion formation in the heart. In the initial set of simulations, designed to evaluate the effect of intrapericardial fluid on myocardial and lung damage, the irrigated fluid temperature was set at 21 °C, assuming this to be a reasonable approximation of ambient temperature in the interventional suite. Additional simulations were performed using irrigation fluid temperatures ranging from 5 °C to 37 °C (core body temperature). In the absence of epicardial fat, reducing the irrigation temperature to 5 °C resulted in a 50% reduction in lung damage volume compared to damage provoked with 37 °C irrigation. With a 1 mm layer of epicardial fat, a 42% reduction was still observed under the same conditions. In contrast, the effect on myocardial lesions was considerably smaller. Without epicardial fat, lesion volume in the myocardium decreased by only 14% when comparing 5 °C and 37 °C irrigation. Interestingly, myocardial lesion depth remained essentially unchanged across irrigation temperatures. In the absence of fat, a depth of 4.6 mm was achieved, while in the presence of 1 mm of fat, lesion depth reached 2.6 mm.

### 4.4. Comparison with Experimental Data

Although our model is based on well-established physical laws and well-characterized properties of biological tissues (see Supplementary Materials), it is important to compare the computational results with experimental data from literature. We compared the results obtained for the case with 8 mm myocardial thickness and no epicardial fat with those reported in three experimental studies based on in vivo models [5,6,15], which reported lesion measurements under ablation settings similar to those simulated by us (see Table 2).

Aryana et al. [5], using 40 W, reported a lesion depth of 5.5 mm in the absence of intrapericardial fluid and 4.5 mm when fluid was present. Our simulations, considering a slightly higher power (50 W), offered lesion depths of 6.1 mm and 5.1 mm for the same two conditions, respectively. While the difference could be explained by the different applied power, the trend is consistent: The presence of intrapericardial fluid is associated with reduced myocardial lesion size. Note that both the experimental [5] and computational results show a 1 mm reduction in lesion depth in the presence of intrapericardial fluid, underscoring the consistency and robustness of this finding across different methodologies. Fenelon et al. [15] reported lesion depths of 6.4 ± 2.1 mm when applying a mean power of 43 W during epicardial ablation. These values are also in reasonable agreement with the computed lesion depths in our study (5.1–6.1 mm). Similarly, D’Avila et al. [6] reported lesion depths of 6.7 ± 1.7 mm under ablation conditions comparable to those simulated in our study. Although slightly higher than our predicted values, the results remain within a reasonable range. Considering that the in vivo experiments were conducted in animal models and myocardial wall thicknesses were not specified, the agreement with our computational findings can be considered satisfactory.

Our results are also consistent with previous studies that found epicardial fat hinders the formation of effective lesions in the myocardium [6,7]. In addition, our study shows that the presence of epicardial fat may also promote thermal damage in structures adjacent to the myocardium, such as the lung. This is due to the low electrical conductivity of fat, which causes more current to flow through the lung (in our model) or any nearby tissue with higher conductivity than fat.

### 4.5. Clinical Implications

The accumulation of fluid within the pericardial space during epicardial procedures is typically considered a potential risk factor due to the possibility of cardiac tamponade. However, our results suggest that excessive intrapericardial fluid may also compromise the efficacy of RF energy delivery. These findings are consistent with those reported by Aryana et al. [5]. Clinically, this suggests that when poor lesion formation is detected during epicardial RFA, one possible contributing factor could be the accumulation of irrigated saline in the pericardial sac. In such cases, drainage of the intrapericardial fluid may be considered not only to avoid cardiac tamponade but also to restore effective energy transmission and improve ablation outcomes. Furthermore, a high irrigation flow rate may contribute to this problem by increasing the amount of fluid retained in the pericardial space. Therefore, in the context of epicardial ablation, a reduced irrigation flow rate might be justified [5]. This practical implication may be particularly relevant in procedures where the electrode–tissue interface is suboptimal or when repeated ineffective pulses raise concerns about insufficient lesion formation.

The literature on the side thermal effect on the lung during epicardial RFA via subxiphoid access with irrigated-tip catheters is limited and mostly anecdotal. D’Avila et al. [6] reported thermal damage to the lung in only one case, while Sacher et al. [31] observed chest pain in most patients, likely due to pericarditis. Dyrda et al. [32], however, found no significant difference in chest pain between patients who underwent only mapping and those who also received ablation. Additionally, Fenelon et al. [15] reported lesions in the parietal pericardium and adjacent lung tissue with both conventional and irrigated-tip catheters, though such damage was absent when using shielded electrodes (70% vs. 0%), supporting the idea that collateral damage is thermally mediated. Given the anatomical variability involved, computational modeling provides a valuable tool to explore the temperature distribution during epicardial ablation and assess potential collateral risks. Our simulations indicate that epicardial RFA may induce thermal damage to the lung, although its clinical significance remains uncertain. These findings may have practical relevance:

**Lung damage volume:** In worst-case scenarios, up to 300 mm^3^ of lung tissue was affected, though this may not result in symptoms or complications.

**Intrapericardial fluid accumulation:** An increase in the PSW from 2.5 to 4.5 mm was associated with reduced thermal damage to the lung and the parietal pericardium, likely due to the increased separation between the ablation electrode and adjacent structures. However, this protective effect comes at the cost of attenuated lesion depth in the myocardium, up to 1 mm shallower, which may be relevant when targeting subepicardial substrates in VT. Moreover, excessive fluid accumulation within the pericardial space may lead to elevated intrapericardial pressure and increase the risk of cardiac tamponade, a critical complication during epicardial ablation procedures.

**Temperature of irrigated saline:** Our findings show that lowering the temperature of the irrigation saline effectively reduces thermal damage in adjacent structures (such as the lung) while only modestly reducing myocardial lesion size. Importantly, lesion depth in the myocardium remains unchanged across irrigation temperatures. This suggests that cooling the irrigation fluid could serve as a protective strategy when nearby structures are at risk, without compromising lesion effectiveness in the myocardium. Nevertheless, further investigation is needed to fully assess the safety and efficacy of this approach and to determine its optimal application in clinical practice.

### 4.6. Study Limitations

In addition to the general limitations of any RF ablation computational modeling, we must highlight the specific limitations of our study below.

#### 4.6.1. Impact of Intrapericardial Fluid Volume

In our simulations, we evaluated the impact of the pericardial space, defined as the distance between the epicardium and pericardiac sac. However, this parameter is difficult to control in clinical practice, as it depends not only on the volumes of irrigation fluid delivered and drained during mapping and ablation but also on the patient’s specific anatomical characteristics. Consequently, achieving precise control over this factor during the procedure remains challenging.

#### 4.6.2. Excluded Ultra-Thin Anatomical Layers and Small Structures

While our model included all major anatomical structures, such as the myocardium, epicardial fat, and lung, other ultra-thin layers forming the interface between these structures, such as the pericardial sac and the pleura, were not included, nor were small structures like the phrenic nerve and coronary arteries, which are vulnerable to damage during epicardial RFA [12,32]. These small structures occupy a minimal volume, and since there is no evidence to suggest that their electrical and thermal properties differ significantly from the surrounding tissue, we think their exclusion has little impact on the overall conclusions.

#### 4.6.3. Irrigation Flow Rate

We assumed a constant irrigation flow rate of 37 mL/min during both the RF pulse and the post-RF period, which is not entirely accurate, since the flow rate is often reduced to minimum values (e.g., 2 mL/min) after the RF pulse is completed in clinical practice. As the thermal damage in the lung increases after the RF pulse, reducing the irrigation flow rate during this period could potentially impact the extent of damage. This effect should be investigated in future studies.

#### 4.6.4. Catheter Orientation and Irrigation Holes Position

In our study, the catheter was considered to be positioned in a horizontal orientation relative to the epicardial surface, with the irrigation holes arranged in a specific spatial configuration. In clinical practice, however, the catheter position can vary between applications due to patient anatomy, access trajectory, or operator preferences. These variations may influence both the distribution of RF-induced heating in the tissues and the cooling effect of the irrigation fluid. Future studies incorporating different catheter orientations and irrigation hole configurations could expand the practical implications of our current results.

#### 4.6.5. Constant Dynamic Viscosity

We assumed that the dynamic viscosity of blood and saline remained constant and independent of temperature, despite it being known that viscosity decreases with increasing temperature. For example, the dynamic viscosity of saline drops from 1.0 mPa·s at 20 °C to 0.3 mPa·s at 90 °C [27]. Complementary simulations accounting for this temperature dependence showed that this only affected lesion size by less than 2.5% for lung lesions and 2% for myocardial lesions, so that while temperature-dependent viscosity could influence fluid mechanics, its effect on the overall lesion size appears to be minimal.

#### 4.6.6. Specific Energy Setting

Although we only simulated a 50 W–30 s RF pulse, in clinical practice different settings may be used, such as lower power levels applied for a long time (1–2 min). While this would result in quantitative differences in the actual lung volume and myocardial damage, there is no reason to think that our qualitative conclusions regarding the impact of the pericardial space and the presence or absence of epicardial fat on lung lesion volume would be invalid.

#### 4.6.7. Single-Lesion Modeling

Our simulations focused on a single RF application. However, epicardial RFA in clinical practice typically involves delivering multiple and contiguous lesions to ensure adequate substrate modification. The cumulative thermal effects, potential tissue preconditioning, and overlapping lesion zones resulting from repeated RF applications were not captured in this model. Therefore, while our findings are informative for understanding the mechanisms involved in individual lesion formation, they may not fully represent the complex thermal dynamics encountered during full procedural workflows.

#### 4.6.8. Effect of the Beating Heart

Our model considered a non-changing geometry, in which the width of the pericardial space remained fixed during ablation. However, due to the heartbeat, it is reasonable to assume that this width will change (even some millimeters), and this could impact the results. Although in a previous computational study on endocardial RF ablation, we observed that the effect of heartbeat had little impact on RF lesion size [33], the epicardial ablation scenario could be different, especially with regard to damage to adjacent structures.

Although all these limitations should be considered when interpreting the results, overall the model provides valuable insights into the thermal effects during epicardial RFA and the potential risks to surrounding tissues, particularly the lung. Further studies are needed to refine these models and explore the clinical relevance of the findings.

## 5. Conclusions

The accumulation of intrapericardial fluid plays a critical role in epicardial ablation outcomes. While it may offer some thermal protection to adjacent structures, it substantially reduces energy transmission to the myocardium, compromising lesion formation. Epicardial fat also impairs lesion formation due to its insulating properties. Finally, reducing the temperature of the irrigation fluid appears to be a simple and potentially valuable strategy to limit collateral thermal damage without compromising lesion depth in the myocardium.

## Figures and Tables

**Table 1:** Physical characteristics of tissues and materials of the elements used in the models at 37 °C [26,27,30].

Element/Material	*ρ* kg/m^3^	*k* W/(m·K)	*c* J/(kg·K)	*σ* (S/m)	*μ* (Pa·s)
Cardiac chamber/blood	1050	0.52	3617	0.748	0.0033
Cardiac wall/myocardium	1081	0.56	3686	0.281	-
Visceral fat/adipose tissue	911	0.21	2348	0.044	-
Lung/pulmonary tissue *	722	0.39	3890	0.215	-
Pericardial sac/irrigation fluid (0.9% NaCl)	1009	0.6	3670	1.44	0.001
Electrode/Pt-Ir	21,500	71	132	4.6×10^6^	-
Catheter/polyurethane	1440	23	1050	10^5^	-

*ρ*: density; *k*: thermal conductivity; *c*: specific heat; *σ*: electrical conductivity (at 37 °C); *μ*: dynamic viscosity. * Mean between inflated and deflated.

**Table 2:** Comparison between experimental and computer results.

		Power (W)	Lesion Depth (mm)	Lesion Volume (mm^3^)
Computer results		50	5.1–6.1	130–262
Aryana et al. [5]	Without intrapericardial fluid	38.9 ± 0.9	5.5 ± 0.52	177.7 ± 42.7
	With intrapericardial fluid	39.1 ± 0.9	4.5 ± 0.59	100.7 ± 26.0
Fenelon et al. [15]		43.0 ± 6.1	6.4 ± 2.1	Not reported
D’Avila et al. [6]		44.8 ± 6.8	6.7 ± 1.7	Not reported

## Data Availability

No new data were created or analyzed in this study.

## Supplementary Materials

The following supporting information can be downloaded at: https://www.mdpi.com/article/10.3390/jcdd12080283/s1, Figure S1. Lesion volumes in the lung and myocardium as the size of the entire model (S) increases [26,27,30,34,35,36,37,38].
