# Supplementary material for: Impact of Intrapericardial Fluid on Lesion Size During Epicardial Radiofrequency Ablation: A Computational Study
Source: J Cardiovasc Dev Dis. 2025 Jul 24;12(8):283. doi: 10.3390/jcdd12080283 (PMC12387105; doi:10.3390/jcdd12080283)
Supplement: Supplementary file 1 [file jcdd-12-00283-s001.zip › jcdd-3732669-supplementary.pdf]

## Supplementary material

*S1. Verification of the computer model*

A sensitivity analysis was performed to determine a suitable outer dimension *S* (see Figure 2 in the manuscript). This analysis was conducted using a representative case with an 8-mm myocardium, a 1-mm fat layer, and a 3.5-mm pericardial sac, as these values lie in the mid-range of the parameters varied in the study. Lesion volumes in the myocardium and lung were evaluated as *S* varied from 40 to 150 mm. The results indicated that lesion sizes stabilize as the model size increases (see Figure 1S). Specifically, for $S \geq 120$ mm, changes in lesion volume were negligible (<0.6 mm³). Therefore, $S = 120$ mm was selected as the most appropriate model size for the simulations, providing a suitable balance between accuracy and computational efficiency.

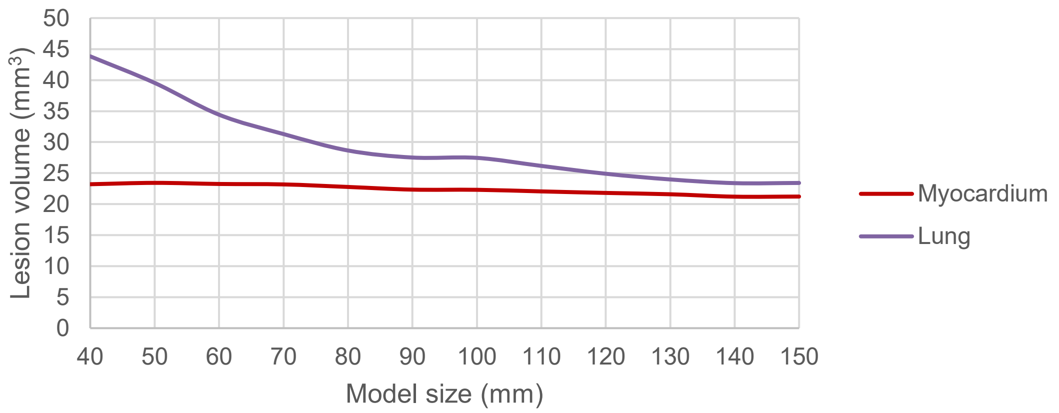


**Figure S1**. Lesion volumes in the lung and myocardium as the size of the entire model (S) increases.

*S2. Governing equations and boundary conditions*

The RF pulse was simulated by solving a coupled electrical-thermal-fluid dynamics problem, with thermal lesion formation computed using the Arrhenius model. During RFA, an electric circuit is established between the catheter

electrode and a dispersive patch placed on the patient’s skin, converting energy losses into heat. This heat increases the temperature, particularly in regions where current density is highest. Heat transfer occurs through conduction between materials, while in fluid-filled subdomains such as blood and the pericardial fluid, convective dissipation also plays a significant role.

The temperature distribution was computed using the heat transfer equation for solids, incompressible fluids, and biological tissues according to [34]:

$$\rho c \frac{\partial T}{\partial t} + \rho c \vec{u} \cdot \nabla \mathrm{T} = \nabla \cdot (k \nabla \mathrm{T}) + Q_{RF} + Q_{P} + Q_{met} \quad (1)$$

where $\rho$ is density (kg/m$^3$), $c$ is specific heat (J/kg·K), $T$ is absolute temperature (K), t is time (s), $\vec{u}$ is velocity vector, $k$ is thermal conductivity (W/m·K), $Q_{RF}$ is the heat source caused by RF power (W/m$^3$), $Q_{met}$ is the metabolic heat generation (W/m$^3$), and $Q_p$ is the heat loss caused by blood perfusion (W/m$^3$) according to:

$$Q_p = \rho_b \cdot c_b \cdot \omega_b \cdot (T_b - T) \quad (2)$$

where $\rho_b$, $c_b$, $\omega_b$ and $T_b$ are blood density (kg/m$^3$), blood specific heat (J/kg·°C), blood perfusion rate (s$^{-1}$), and blood temperature (37 °C), respectively. Both $Q_p$ and $Q_{met}$ were considered to be negligible except for the myocardium and the lung, where $\omega_b$ was 0.018 and 0.002 s$^{-1}$, and $Q_{met}$ was 39.45 and 6.21 W/kg, respectively [26]. $Q_p$ and $Q_{met}$ were only considered when the tissue was below 55 °C, i.e. were set to zero to simulate the tissue death once the temperature this lethal threshold [35]. The temperature at the beginning of the pulse was set at 37 °C. As the model has two fluid inlets, irrigation fluid and blood stream, the temperature for those inlet fluids were set at 21 °C and 37 °C, respectively.

The electrical problem was solved using the Laplace's equation, which governs current conservation in a medium without internal sources:

$$\nabla \cdot (\sigma(T) \nabla \Phi) = 0 \quad (3)$$

where $\sigma$ is the electric conductivity (S/m) and Φ is the voltage (V). The electric field E (V/m) was then computed as:

$$|\vec{E}| = -\nabla \Phi$$

The electromagnetic loses $Q_{RF}$ (W/m$^3$), representing the heat generated by the induced current, were calculated as:

$$Q_{RF} = \sigma \cdot |\vec{E}|^2 \quad (4)$$

To simulate a 50 W RF pulse, an electrical boundary condition of constant power at 20 W was applied. This adjustment accounts for two factors: 1) the model represents only half of the volume due to the symmetry plane, and 2) the model is constrained to a limited domain, excluding the dissipation of RF energy in tissues distant from the heart and below the dispersive patch, which accounts for approximately 20% of the total energy delivered [36]. The model's external boundaries were set to 0 V to simulate the position of the dispersive patch in the distance.

Fluid mechanics for irrigation fluid and blood stream was solved using the Navier-Stokes equations (Eq. (5)), assuming constant viscosity (as mentioned earlier), incompressible flow (Eq. (6)), and negligible effects of gravity.

$$\rho \frac{\partial \vec{u}}{\partial t} + \rho(\vec{u} \cdot \nabla)\vec{u} = \nabla \cdot \left[-pI + \tau_{ij}\right] \quad (5)$$

$$\rho(\nabla \cdot \vec{u}) = 0 \quad (6)$$

where $\rho$ is density (kg/m$^3$), $\vec{u}$ is velocity vector (m/s), $t$ is time (s), $p$ is pressure (Pa), and $\tau_{ij}$ is the stress tensor (Pa) [37].

The Irrigation fluid was introduced into the pericardial sac through the holes located in the catheter electrode and dispersed throughout the pericardial space. The blood flow was modeled as a laminar flow that runs through the corresponding space in a single direction. It has the entrance on one side of the model, and the exit on the opposite side. The average blood velocity has been set to 24.5 cm/s [38].

Thermal damage in the myocardium and lung was assessed using the Arrhenius equation:

$$\frac{\partial \alpha}{\partial t} = (1 - \alpha) \cdot Ae^{-\Delta E/_{RT}} \quad (7)$$

where $\alpha$ represents the extent of thermal damage, $A$ = $7.39 \cdot 10^{39}$ $s^{-1}$ is the frequency factor, $\Delta E$= $2.577 \cdot 10^5$ J/mol is the activation energy, $R$ = 8.314 (J/mol·K) is the universal gas constant, and $T$ is the absolute temperature in Kelvin. The term (1−α) represents the fraction of undamaged tissue [34,38].

*S3. Temperature dependent viscosity*

Additionally, a comparison was made between a simulation in which the irrigation fluid (which is the fluid expected to reach the highest temperature) had a temperature-dependent viscosity and another in which its viscosity remained constant. This was done for the specific case of 8 mm myocardium, 1 mm fat layer and 3 mm pericardial sac. The differences in lesion size were 0.4% for the myocardium (58.1 $mm^3$ vs. 58.4 $mm^3$) and 0.8% for the lung (148.5 $mm^3$ vs. 147.2 $mm^3$), which can be considered negligible. Therefore, using a constant viscosity instead of a temperature-dependent value is a reasonable approximation which allows reducing the computational cost. Henceforth, a constant viscosity value of 0.001 Pa·s [27] corresponding to water at 21 ºC was

used for the irrigation fluid, while a viscosity value of 0.0033 Pa·s [30] corresponding to blood at 37 ºC, was set.

## References for the supplementary material

34. Berjano, E.J. Theoretical modeling for radiofrequency ablation: State-of-the-art and challenges for the future. *Biomed. Eng. OnLine* **2006**, *5*, 24. https://doi.org/10.1186/1475-925X-5-24.

26. Hasgall, P.A.; Di Gennaro, F.; Baumgartner, C.; Neufeld, E.; Lloyd, B.; Gosselin, M.C.; Payne, D.; Klingenböck, A.; Kuster, N. *IT'IS Database for Thermal and Electromagnetic Parameters of Biological Tissues, Version 4.1*; IT'IS Foundation: Zürich, Switzerland, 2022. Available online: https://itis.swiss/virtual-population/tissue-properties/overview/ (accessed on 5 May 2024).

35. Haines, D.E. Letter by Haines regarding article, "Direct measurement of the lethal isotherm for radiofrequency ablation of myocardial tisssue". *Circ. Arrhythm. Electrophysiol.* **2011**, *4*, e67; author reply e68. https://doi.org/10.1161/CIRCEP.111.965459.

36. Irastorza, R.M.; Gonzalez-Suarez, A.; Pérez, J.J.; Berjano, E. Differences in applied electrical power between full thorax models and limited-domain models for RF cardiac ablation. *Int. J. Hyperth.* **2020**, *37*, 677–687. https://doi.org/10.1080/02656736.2020.1777330.

37. White, F.M.; Majdalani, J. *Viscous Fluid Flow*, 4th ed.; McGraw-Hill Higher Education: New York, NY, USA, 2021; ISBN 9781260597806.

38. Parés, C.; Berjano, E.; González-Suárez, A. Effect of intracardiac blood flow pulsatility during radiofrequency cardiac ablation: Computer modeling study. *Int. J. Hyperth.* **2021**, *38*, 316–325. https://doi.org/10.1080/02656736.2021.1890240.

27. Haynes, W.M. *CRC Handbook of Chemistry and Physics*, 95th ed.; CRC Press : Boca Raton, FL, USA, 2014; ISBN 9781315380476.

30. Guyton, A.C.; Hall, J.E. *Textbook of Medical Physiology*, 20th ed.; Saunders : Philadelphia, PA, USA, 2011; ISBN 9781416045748.
